# Additive Manufacturing Using Multi-Materials: Materials, Processes, and Applications

**DOI:** 10.3390/polym18091045

**Published:** 2026-04-25

**Authors:** André F. V. Pedroso, Francisco J. G. Silva, Alexandra Gavina, Isabel Figueiredo, Ana Almeida Silva

**Affiliations:** 1 CIDEM, ISEP, Polytechnic of Porto, Rua Dr. António Bernardino de Almeida, 4249-015 Porto, Portugal; afvpe@isep.ipp.pt (A.F.V.P.); alg@isep.ipp.pt (A.G.); ipf@isep.ipp.pt (I.F.); 1201039@isep.ipp.pt (A.A.S.); 2 Department of Mechanical Engineering, Faculty of Engineering, University of Porto, Rua Dr. Roberto Frias, 400, 4200-465 Porto, Portugal; 3 Associate Laboratory for Energy, Transports and Aerospace (LAETA-INEGI), Rua Dr. Roberto Frias, 400, 4200-465 Porto, Portugal

**Keywords:** additive manufacturing, multi-material fabrication, advanced materials, manufacturing processes, functional integration, industrial applications

## Abstract

Additive manufacturing (AM) has transformed traditional manufacturing by enabling the fabrication of complex geometries and functional components that are difficult or impossible to produce using conventional techniques. Recent advancements have expanded AM capabilities through the integration of multi-material systems, allowing for enhanced performance, customisation, and functionality of manufactured parts. Despite rapid development, there is a limited consolidated understanding of the processes, material combinations, and practical implications of multi-material additive manufacturing (MMAM) across different application domains. This study aims to provide a comprehensive overview of general additive manufacturing processes, with a particular focus on the evolution and implementation of multi-material fabrication techniques. The review draws upon publicly available scientific literature to analyse various AM technologies, material pairing strategies, and process parameters. Comparative analysis is conducted between the additive and conventional manufacturing approaches to highlight advantages and limitations. The findings reveal significant progress in material compatibility, interface bonding, and process integration, enabling the production of multifunctional and performance-optimised components. Diverse applications are identified across aerospace, biomedical, and industrial sectors. MMAM represents a critical advancement in modern manufacturing, offering expanded design freedom and functional integration. Continued research is essential to address the remaining challenges in material compatibility, scalability, and process standardisation.

## 1. Introduction and Process Contextualization

Additive manufacturing (AM) is a process that includes all the different technologies that produce parts through the successive addition of layers [1,2]. The roots of contemporary AM trace back to the mid-twentieth century, marked by a 1951 patent by Otto John Munz. This patent can be seen as the inception of the modern stereolithography technique. Munz devised a system for selectively exposing a transparent photo emulsion layer-by-layer, where each layer received exposure corresponding to a cross-section of an object [3,4]. But why is it better, or why did AM start to be used? There are three main differences between conventional manufacturing and AM: time, waste, and the layout or occupied space.

**Figure 1:**
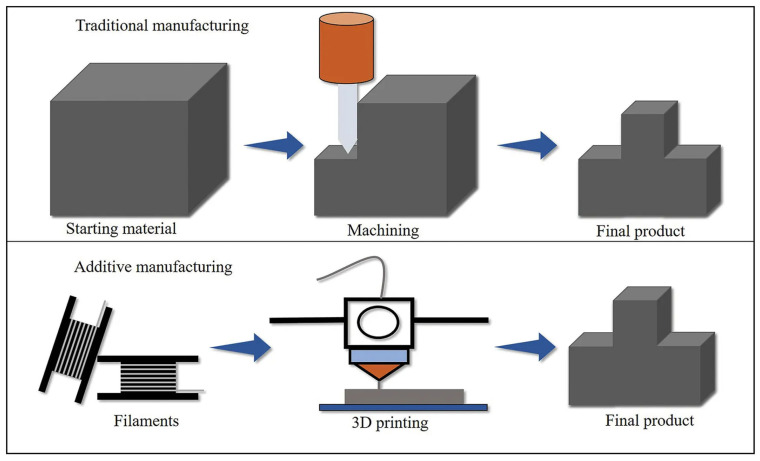
Conventional subtractive manufacturing vs AM [5].

Starting with the time spent, in traditional manufacturing it is necessary to spend multiple days or even weeks to obtain a complete piece when you have a raw material. When AM is used, only a few hours are needed to complete the entire process. This happens because conventional manufacturing contains different processes to obtain a finished piece. First, processes like melt and cast are used to give the initial form. This process alone takes a considerable amount of time because the raw material needs to be heated to its fused temperature, and then it must cool down until it returns to a solid state. Then it can be used, formed like an extrusion, or pultrusion, to get closer to the desired shape and to modify some properties. After that, it is used in a machining process to remove excess material, and it could be necessary to paint or polish the piece. In the end, it could be necessary to join some parts using a process like welding. Figure 1 is a schematic of this situation. When AM is chosen, most of this process is no longer needed.

**Figure 2:**
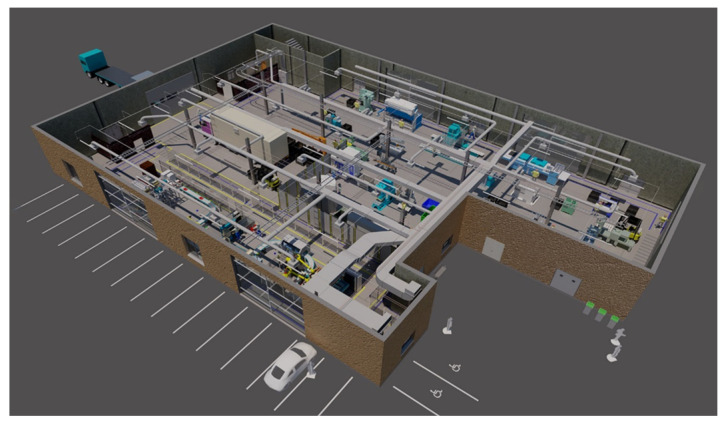
Conventional manufacturing factory.

Moving on to the topic of waste, to obtain the same object, conventional manufacturing will produce a lot more waste compared to AM. This happens because the conventional methods are associated with subtractive manufacturing; in other words, it all starts with a solid piece, and the material is removed until the desired shape is achieved. When AM is used, as the name implies, it is a process where the material is added layer-by-layer to make an object. This way, the resulting waste is almost non-existent [6,7]. To conclude the comparison, a different type of manufacturing implies different factory layouts. Since traditional manufacturing includes a lot of different process that implies more machines, which leads to more space occupied (Figure 2).

**Figure 3:**
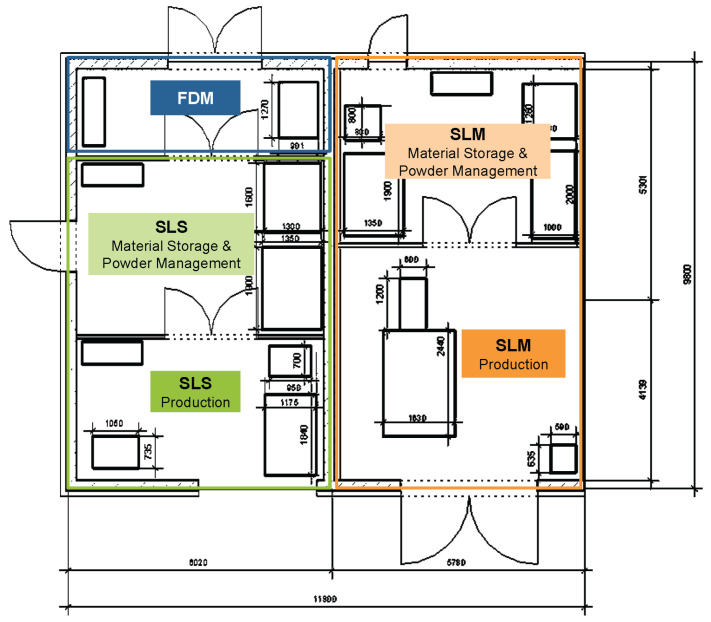
Model Factory for AM [8].

In AM, just one machine is capable enough to complete the work. In Figure 3, it is possible to understand that in a smaller space, different AM machines can be placed.

When multi additive materials are applied, it is necessary to consider the orientation of the layers of the different materials [2]. There are two distinct approaches employed to define material modelling components: the first is grounded in the Powder Bed Fusion (PBF) machine coordinate system, while the second utilises the material gradient within the part structure. The coordinate system for AM systems is outlined in the ISO 17295:2003 standard (the older ISO/ASTM 52921:2013 standard). The perspective based on layers aligns with this definition, characterising the material transition between layers as a 2D structure with a material shift along the z-axis. The transition of material within and between layers is then conceptualised as a 3D structure [9,10,11,12].

Although numerous studies and reviews have examined specific MMAM processes, individual material combinations, or targeted application domains, a comprehensive cross-platform synthesis remains comparatively limited. Existing literature frequently focuses on single process categories (e.g., directed energy deposition, powder bed fusion, or extrusion-based systems) without systematically comparing multi-material strategies across different AM technologies within a unified analytical framework.

This study provides a comprehensive and integrative synthesis of recent advancements in MMAM, encompassing innovations in processing platforms, machine architectures, and material system combinations. By systematically analysing developments across major AM technologies, this review goes beyond descriptive reporting and adopts a materials-science-driven perspective. Emphasis is placed on interfacial phenomena, phase stability, solidification behaviour, residual stress evolution, and long-term reliability, thereby linking process selection, interface integrity, and technological maturity within a coherent comparative framework.

Furthermore, this review introduces comparative performance assessment and technology maturity mapping of MMAM processes, enabling clearer identification of industrial readiness, capability limitations, and future research directions.

This paper presents a systematically structured and critically informed investigation into the domain of Multi-Material Additive Manufacturing (MMAM) [13], articulated across seven interlinked sections designed to provide conceptual depth, methodological transparency, and analytical coherence. Collectively, these sections advance a comprehensive examination of the scientific, technological, and industrial dimensions shaping the contemporary evolution of multi-material AM. Section 1 establishes the contextual and theoretical foundations of the study, situating contemporary AM technologies within the evolving landscape of multi-material fabrication and articulating their scientific, technological, and industrial significance. Section 2 delineates the methodological novelty of the review framework, emphasising the distinguishing features that differentiate the present study from prior systematic and narrative reviews in the field. Section 3 details the research methodology and information sources, providing a transparent account of the data acquisition, screening, and quality-assessment procedures underpinning the review. Section 4 presents the organisational structure of the manuscript, offering a conceptual roadmap to facilitate coherent navigation of the analytical narrative. Section 5 delivers a critical and thematically structured synthesis of the extant literature, systematically examining prior research on multi-material AM through dedicated subsections addressing material systems and processing routes, key process parameters, machine architectures, application domains, technical challenges, and emerging research trajectories. Section 6 advances an integrative discussion, elucidating cross-study relationships, identifying convergent and divergent trends, and extracting overarching patterns that characterise the current state of multi-material AM research and practice. Finally, Section 7 articulates the principal contributions of the present work, clarifying how the study addresses critical gaps in the literature and advances conceptual, methodological, and technological understanding within the domain of multi-material AM.

In the interest of scholarly transparency and professional accountability, the manuscript explicitly acknowledges its inherent limitations, offering a critical reflection on constraints arising from data availability, methodological heterogeneity, and the rapidly evolving technological landscape of multi-material AM. The paper concludes with a rigorous synthesis of principal findings and forward-looking insights, thereby delivering a comprehensive and authoritative perspective on the current state of the field and delineating strategic pathways for future academic inquiry, industrial innovation, and professional practice.

## 2. Method of Research

A systematic and comprehensive literature review was undertaken in accordance with a transparent and replicable research design [14,15] and was explicitly aligned with the Preferred Reporting Items for Systematic Reviews and Meta-Analyses (PRISMA) 2020 framework (Appendix A), thereby ensuring procedural clarity, methodological rigour, and reproducibility [16]. The evaluation of candidate sources was informed by established quality indicators, including citation impact and the scholarly standing of the publication venues. Data collection was conducted in January 2026, with primary records retrieved from the CrossRef database, reflecting its broader indexing scope. To broaden the search scope and enhance consistency across metadata fields, supplementary indexing platforms, most notably Dimensions.ai, were also consulted. Furthermore, a bespoke MATLAB^®^ algorithm [17] was developed in the R2024a version of the software and employed to automate record consolidation, harmonise cross-database outputs, and systematically identify and remove duplicate entries.

### 2.1. Search Strategy and Information Sources

Building upon the PRISMA-aligned review framework described above, the literature search strategy was designed to explicitly identify studies focused on MMAM rather than general AM research. To ensure thematic relevance, the search combined three conceptual groups of keywords: (1) AM processes: “additive manufacturing”, “3D printing”, “laser powder bed fusion”, LPBF, SLM, DED, LENS™, “material extrusion”, Fused Deposition Modelling (FDM), Fused Filament Fabrication (FFF), Direct Ink Writing (DIW), “material jetting”, Stereolithography (SLA), and Digital Light Processing (DLP); (2) Multi-material fabrication concepts: “multi-material”, “multi material”, multi-material, “functionally graded material”, FGM, “material gradient”, “graded structure”, “multi-nozzle”, “multi-feed deposition”, “material switching”, and “in situ material mixing”; and (3) Interface and performance-related aspects: interface, bonding, adhesion, compatibility, interfacial behaviour, material transition, residual stress, and delamination.

The Boolean search structure followed the general formulation:

(“Additive Manufacturing” OR “3D Printing”)
AND (“Multi-material” OR “Functionally Graded” OR “Material Gradient”)
AND (interface OR bonding OR compatibility OR performance)

Searches were applied to the title, abstract, and keywords fields, and the following eligibility filters were applied:

- Publication period: 1998–2025
- Document type: peer-reviewed journal articles and review papers
- Language: English

These restrictions were introduced to minimise the inclusion of studies addressing single-material AM or unrelated materials research domains.

### 2.2. Study Selection and Screening

The systematic identification and selection of studies conformed to the PRISMA 2020 framework, thereby ensuring procedural transparency and methodological reproducibility throughout the review process. The initial database query across Web of Science, Scopus, and CrossRef yielded a total of 367,617 records spanning the 1998–2025 period. The initially large number of retrieved records reflects the broad indexing coverage of CrossRef and associated metadata repositories, which include multiple database entries, early-access versions, and publisher-level indexing duplicates subsequently removed during automated consolidation. Following combined automated and manual deduplication using the bespoke MATLAB^®^ routine, 22,159 unique records were retained for preliminary screening. Following automated deduplication, screening and eligibility decisions were performed according to predefined inclusion and exclusion criteria established before study selection to minimise subjective bias and ensure methodological consistency. This substantial reduction primarily resulted from cross-database overlap and publisher-level duplication across indexing platforms. Since this structured framework, a coherent and hierarchically structured set of Research Questions (RQs) was formulated to guide the systematic examination and synthesis of the selected body of literature.

Subsequently, titles, abstracts, and keywords were evaluated to determine their relevance to multi-material AM, with particular emphasis on material integration, interfacial behaviour, process reliability, functional performance, and long-term durability of multi-material components. Studies lacking methodological rigour, failing to address multi-material systems, or falling outside the scope of interface engineering, hybrid manufacturing, process optimisation, or performance evaluation were excluded. This screening stage resulted in 9098 studies progressing to full-text assessment. The majority of excluded studies at this stage addressed single-material AM despite initial keyword relevance.

To ensure methodological consistency, inclusion and exclusion criteria were defined before database querying, and the screening procedure followed a structured multi-stage protocol aligned with PRISMA 2020 recommendations, thereby reducing subjective bias in study selection.

Following the application of clearly defined inclusion and exclusion criteria, centred on experimental robustness, industrial relevance, and theoretical coherence, a final corpus of 151 peer-reviewed publications was retained. As two journals accounted for a disproportionately high volume of contributions, and to mitigate potential source bias, the most influential articles were selected based on citation impact. This curated set of studies constitutes the empirical and conceptual foundation of the present comprehensive review. This multi-stage selection procedure ensured that only high-quality contributions addressing multi-material AM processes, material compatibility, interface behaviour, machine architectures, process parameter optimisation, and functional performance of printed components were retained. The final corpus, therefore, represents the most scientifically robust and industrially relevant body of work elucidating the development, performance, and application of multi-material AM systems over the past decade.

A recurring challenge encountered during the literature review concerned the non-standardised nomenclature used to describe materials and processes across the field. Despite the availability of internationally recognised classification systems, identical materials and manufacturing approaches are frequently reported under multiple designations. For instance, comparable polymer blends, metal alloys, or ceramic composites are often referenced using trade names, compositional shorthand, or process-specific terminology rather than standardised material identifiers. Similar inconsistencies arise in the naming of AM processes, such as the interchangeable use of proprietary and generic terms (e.g., LPBF, SLM, or DMLS). This lack of terminological uniformity necessitated systematic cross-verification of material compositions, processing routes, and standards during the screening and categorisation of studies to avoid misinterpretation of reported material behaviour and performance. Furthermore, linguistic variation between American and British English (for example, “modelling” versus “modelling”) introduced additional, albeit minor, sources of inconsistency within the bibliographic records.

The formulation of precise RQs constitutes a central element of any systematic literature review, as it defines the conceptual scope and analytical direction of the investigation. To ensure internal coherence and alignment with state-of-the-art developments in multi-material AM, the Population, Intervention, Comparison, Outcome, and Context (PICOC) framework was adapted as follows:

- Population: Experimental, numerical, and review studies addressing the design, fabrication, interfacial behaviour, mechanical, thermal, electrical, or functional performance of multi-material additively manufactured components, encompassing polymer–polymer, metal–metal, metal–ceramic, and metal–polymer systems across a range of AM technologies.
- Intervention: Multi-material fabrication and interface-engineering strategies, including functionally graded materials, in situ material mixing, multi-nozzle and multi-feed deposition systems, hybrid additive–subtractive manufacturing approaches, laser-based material modulation, and post-processing or interlayer modification techniques aimed at enhancing bonding, performance, and reliability.
- Comparison: Comparisons between single-material and multi-material components, between discrete and graded material transition architectures, and across different AM process classes and machine configurations, with emphasis on material compatibility, interface quality, and functional performance.
- Outcomes: Improvements in interfacial strength, mechanical integrity, thermal stability, electrical or functional performance, dimensional accuracy, process reliability, and long-term durability, as well as the identification of emerging technological trends and unresolved scientific and engineering challenges in MMAM.
- Context: Peer-reviewed academic and industrial publications addressing MMAM technologies, materials systems, digital design and modelling approaches, process integration strategies, and application-driven performance evaluation, published between 1998 and 2025.

Based on this framework, a coherent and hierarchically structured set of Research Questions was formulated to guide the systematic extraction, critical evaluation, and synthesis of knowledge from the retained body of literature. These RQs are presented in Table 1 of the manuscript.

**Figure 4:**
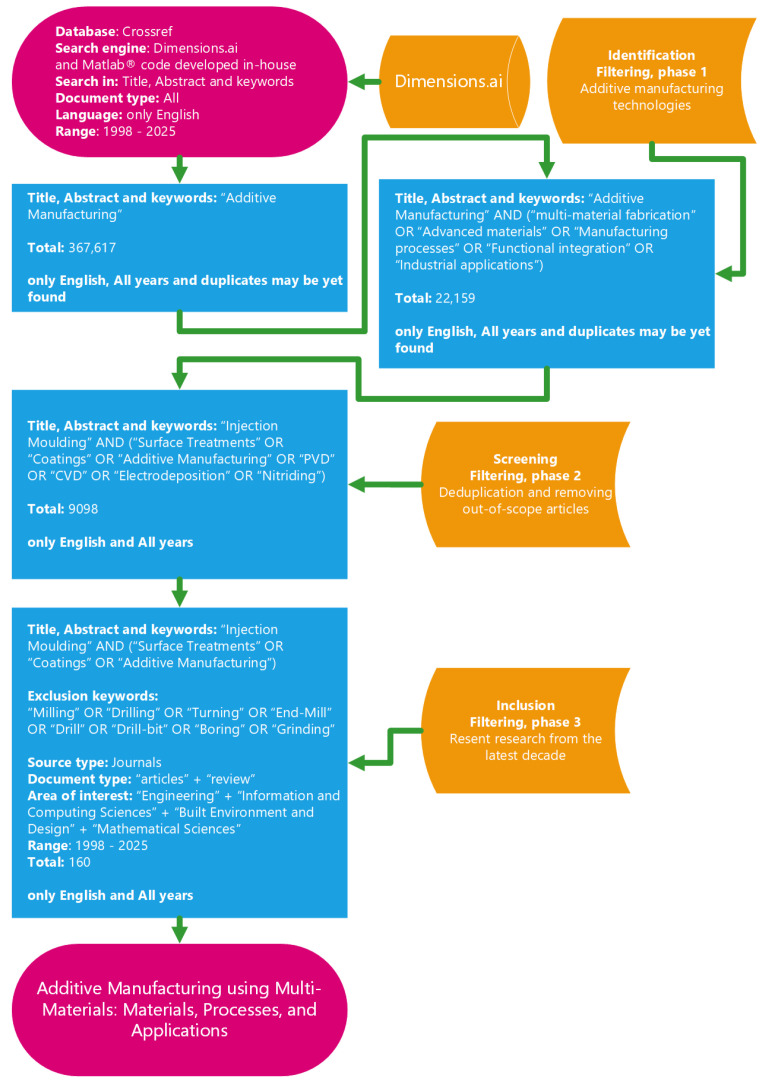
PRISMA-Based Three-Level Screening and Selection Protocol for AM searched articles.

The review encompassed peer-reviewed publications issued between 1998 and 2025, with particular emphasis on the most recent decade of scholarship addressing the development, performance, and industrial deployment of multi-material AM systems. The literature search, schematized in Figure 4, was conducted using structured Boolean queries incorporating the keyword combinations of “Additive Manufacturing” and (“multi-material fabrication” or “Advanced materials” or “Manufacturing processes” or “Functional integration” or “Industrial applications”), thereby selectively capturing contributions directly pertinent to material compatibility, interfacial behaviour, process integration strategies, and the functional performance of multi-material AM technologies.

**Figure 5:**
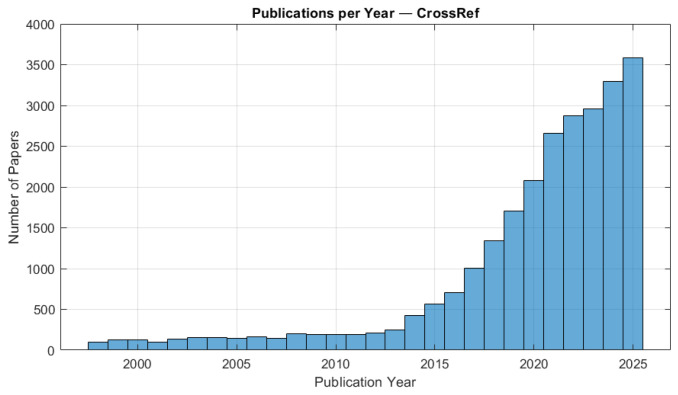
Publications per year as queried to CrossRef.

Across the CrossRef repository, a rapid increase in research output is evident from 2013 to 2025 (Figure 5). The continuous growth of the subject demonstrates academic and industrial interest in surface treatments, coating technologies, and AM approaches aimed at improving the injection mould tool longevity and operational performance.

### 2.3. Study Selection and Quality Assessment (QA)

The assessment of methodological quality represents a critical stage in any systematic literature review, as it establishes the evidential foundation upon which the credibility, validity, and interpretative robustness of the ensuing synthesis depends. In the present review, a QA framework was developed to evaluate the rigour, transparency, and practical relevance of each selected study through a structured, multi-dimensional evaluative approach. The framework comprised four equally weighted categories, each addressing a distinct dimension of methodological integrity and scholarly contribution. The assessment criteria were adapted from established systematic review protocols and operationalised using a three-point ordinal scale, wherein “*Yes*” = 1, “*Partly*” = 0.5, and “*No*” = 0.

The four QA categories were defined as follows:

- Research Design and Theoretical Foundation (25%)—assessing the clarity of research aims, the appropriateness of the methodological approach, and the coherence of the underlying theoretical and conceptual framework.
- Technical Scope and Materials/Process Definition (25%)—evaluating the precision with which multi-material systems, material combinations, interface architectures, and AM processes are characterised, including the adequacy and reproducibility of reported experimental and processing parameters.
- Methodological Soundness and Analytical Precision (25%)—examining the transparency of data acquisition and processing procedures, the robustness of validation and verification methods, and the consistency and rigour of analytical interpretation.
- Practical and Industrial Relevance (25%)—determining the extent to which the reported findings demonstrate applicability to real-world MMAM scenarios, including implications for scalability, process stability, component performance, and industrial feasibility.

**Figure 6:**
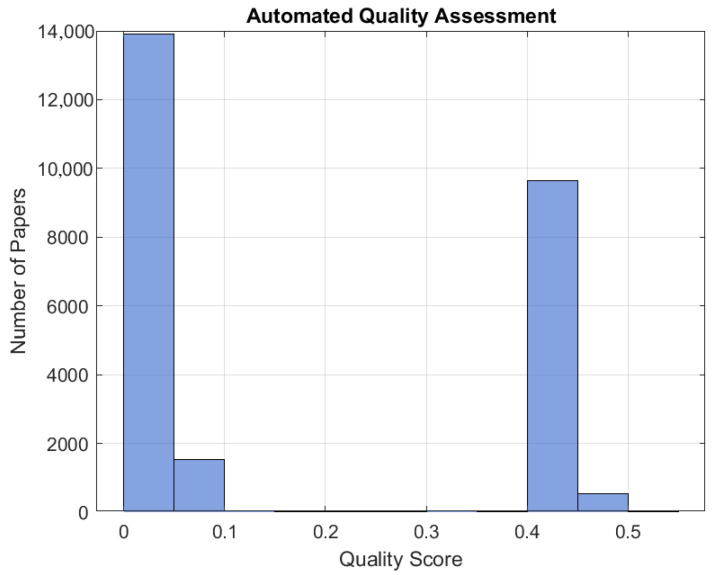
QA of the gathered articles.

To ensure the integrity and reliability of the final synthesis, only studies achieving a QA score exceeding 50%, corresponding to a total score greater than 5 out of 10, were retained for inclusion, as illustrated in Figure 6. A minimum QA threshold of 50% was adopted to avoid a premature exclusion of the studies addressing emerging MMAM technologies, where methodological reporting may vary across experimental and review contributions. Rather than functioning as a strict quality ranking mechanism, the QA procedure was designed to ensure baseline methodological reliability while preserving technological diversity within the analysed literature.

Studies obtaining scores close to the threshold were therefore subjected to additional qualitative verification to confirm their technical relevance, methodological consistency, and alignment with the objectives of the review. Consequently, the final corpus comprised contributions demonstrating acceptable methodological rigour, empirical significance, and conceptual clarity, particularly regarding material compatibility, interface performance, process integration, and functional reliability of MMAM systems. Studies obtaining QA scores within the borderline interval (50–60%) were re-examined through full-text evaluation by the authors to verify the clarity of process description, material definition, and reported outcomes before final inclusion.

The identification and selection of relevant studies followed a three-stage screening protocol consistent with the PRISMA 2020 framework, ensuring a systematic and transparent progression from initial retrieval to final inclusion. In the first stage, all search outputs were consolidated, and duplicate records were removed through a combination of automated and manual verification using the bespoke MATLAB^®^ routine.

**Figure 7:**
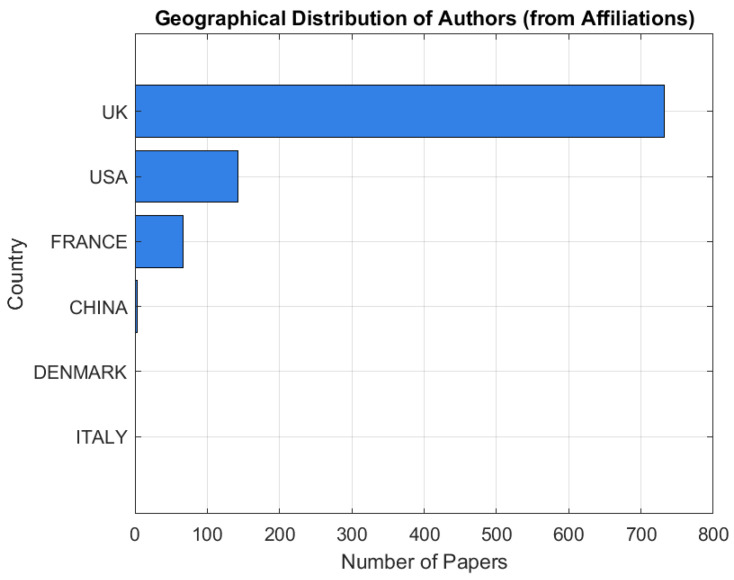
Geographical distribution of authors by institutional affiliation.

As illustrated in Figure 7, the geographical distribution of authorship and institutional affiliation across the reviewed studies indicates a pronounced concentration of research activity within Europe, with the United Kingdom emerging as a leading contributor to the scholarly literature on multi-material AM processes, material integration strategies, and interface engineering. A substantial proportion of publications also originate from the United States, reflecting the global relevance and strong industrial engagement associated with the development and deployment of MMAM technologies.

This spatial distribution is noteworthy, as it indicates that research leadership in MMAM is geographically diffused rather than concentrated within a limited set of countries traditionally associated with established strengths in manufacturing, precision engineering, and materials science. This dispersion underscores the increasingly international and collaborative character of contemporary research efforts directed towards advancing material integration, interface reliability, process scalability, and the functional performance of multi-material additively manufactured systems.

### 2.4. Descriptive Analysis and Taxonomic Synthesis

The descriptive synthesis of the final corpus reveals a heterogeneous and rapidly evolving research landscape, characterised by an intensifying convergence of materials science, machine innovation, and digital process integration in the pursuit of robust, multifunctional, and industrially deployable MMAM systems. The reviewed body of work was systematically classified according to its technological orientation, application domain, and depth of experimental and methodological integration, thereby delineating the principal axes along which contemporary MMAM research is advancing. The literature coalesces around two dominant and interdependent domains:

1. Multi-material Systems and Interface Engineering Technologies: Encompassing polymer–polymer, metal–metal, metal–ceramic, and metal–polymer material architectures, including functionally graded materials, voxel-based material distributions, in situ material mixing strategies, and interlayer modification approaches aimed at enhancing interfacial adhesion, mechanical integrity, thermal compatibility, and multifunctional performance.
2. Process Integration and Manufacturing Innovation Technologies: Incorporating hybrid additive–subtractive manufacturing platforms, multi-nozzle and multi-feed deposition architectures, laser-based material modulation strategies, advanced digital design and simulation frameworks, and scalable machine configurations intended to improve process stability, geometric fidelity, and industrial throughput.

This classification underscores the intrinsically multidisciplinary and multi-scale character of contemporary MMAM research, wherein materials engineering, process physics, digital design methodologies, and system-level manufacturing strategies converge to address the complex thermomechanical, chemical, and structural phenomena governing multi-material interface behaviour and long-term component reliability. Application mapping across the literature reveals four dominant and interrelated thematic trajectories:

1. Interfacial Integrity and Failure Mitigation: Focusing on strategies to suppress delamination, brittle phase formation, residual stress accumulation, and interfacial cracking arising from thermal expansion mismatch, compositional discontinuities, and heterogeneous microstructural evolution.
2. Thermal, Mechanical, and Functional Performance Optimisation: Addressing the development of graded material transitions, architected material distributions, and hybrid reinforcement strategies designed to enhance load-bearing capacity, thermal stability, electrical conductivity, and multifunctional integration.
3. Process Control, Scalability, and Digital Workflow Integration: Examining the role of process parameter optimisation, real-time monitoring, material switching protocols, and advanced CAD and simulation environments in enabling reproducible, large-scale, and application-ready multi-material production.
4. Manufacturing Efficiency and System-Level Performance: Exploring the influence of machine architecture, build strategy, post-processing pathways, and hybrid manufacturing routes on dimensional accuracy, surface integrity, production throughput, and lifecycle performance.

Across the reviewed studies, several quantifiable performance gains are consistently reported:

1. Substantial enhancements in interfacial strength and structural integrity, leading to improved mechanical reliability and extended functional lifespan of multi-material components.
2. Improved thermal compatibility and stress redistribution, resulting in reduced residual stress accumulation and enhanced dimensional stability.
3. Advances in multifunctional integration, enabling the embedding of electrical, thermal, sensing, or actuation capabilities within monolithic, additively manufactured architectures.
4. Increased manufacturability of geometrically complex and architected structures through the adoption of hybrid manufacturing platforms and functionally graded design strategies.
5. Performance evaluation frameworks commonly employ metrics such as interfacial shear and tensile strength, porosity and phase distribution, thermal conductivity, electrical performance, fatigue resistance, and long-term functional stability.

Notwithstanding these advances, the literature consistently identifies persistent and systemic constraints that delimit the full industrial realisation of MMAM technologies. These include variability in interface quality, heterogeneous microstructural development, limited process repeatability, and the formation of undesirable intermetallic or brittle phases at material junctions. Practical impediments—such as material contamination during transitions, constrained recyclability, limited scalability, and deficiencies in commercially available multi-material CAD and simulation platforms—further impede widespread industrial adoption. Moreover, the absence of standardised experimental protocols and qualification frameworks continues to obstruct rigorous cross-study comparison and technology benchmarking.

The review’s three-stage screening methodology commenced with a structured appraisal of titles, abstracts, and keywords to isolate contributions pertinent to multi-material systems, interface engineering, process integration, and functional performance. Subsequent full-text evaluation enforced compliance with predefined inclusion criteria, privileging studies of demonstrable empirical depth, methodological rigour, and industrial relevance. Only peer-reviewed journal articles were retained, while non-English publications, grey literature, and studies lacking analytical transparency were systematically excluded. The resulting dataset, therefore, constitutes a carefully curated and scientifically authoritative foundation underpinning the analytical and interpretative framework advanced in this review.

The review has been retrospectively registered on the Open Science Framework (OSF) (DOI: 10.17605/osf.io/px87b; url: https://osf.io/px87b; accessed on 30 March 2026).

## 3. Literature Review

### 3.1. Materials and Processes

**Figure 8:**
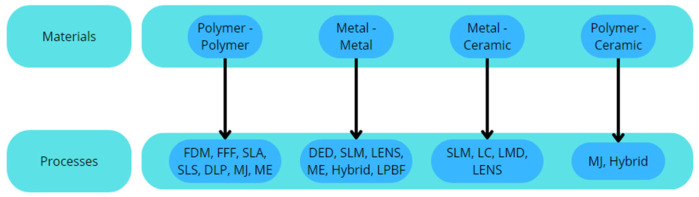
Materials and processes used in MMAM.

In this chapter, it will be possible to understand the different materials used along with their respective AM process [18,19]. Figure 8 demonstrates these different combinations.

Over the past few years, research and investigations have been conducted to test the combination of different materials and processes to improve various properties. Starting with metals and metal alloys, in 2020, Chen et al. [20] did research using 316L steel with CuSn10 through the process of selective laser melting and were able to enhance the heat transfer capacity. Other research was carried out using the same AM process. In the same year, Bartolomeu et al. [21] at the University of Minho and in the Polytechnic Institute of Leiria made an investigation using nickel-titanium alloy and titanium, aluminium, and vanadium alloy as multi-material cellular structures targeting orthopaedic implants, concluding that there is a potential improvement in the relationship mechanics between the bone and implant. Three years before Demir and Previtali [22] proved that there is a growth in hardness using SLM, doing a multi-material consisting of iron, aluminium and silicon alloy. These two researchers also did a study with the same process but using CoCr alloy to produce cardiovascular stents, and the outcomes were that these stents can be allocated as a single-step process and that the SLM productivity can outperform the conventional stent manufacturing scheme, although the AM process to produce cardiovascular stents should be optimised [23].

It is possible to find other studies using metal and metal alloys, but with other AM processes, such as laser engineered net shaping and direct energy deposition. Regarding the process of Laser Engineered Net Shaping (LENS™), in 2010 España et al. [24] studied metallic biomaterials using cobalt-chromium and molybdenum alloy with titanium and aluminium alloy (CoCrMo + Ti6Al4V) to design and fabricate novel structures for load bearing implants achieving an increase of hardness and obtain structures that potentially get rid of the long issues like stress shielding and the deficient interfacial bond between the host tissue. Other work developed by Onuike et al. [25] presents bimetallic structures of Inconel 718 with Ti6Al4V alloy, concluding that it is necessary to use a third material to bond the other two. However, in an additional phase, Cr3C2 was identified and improved the bonding strength, lowered thermal stresses in the interfaces, reaching the conclusion that these structures have a potential application in aerospace, such as launch vehicles’ thermal protection systems.

Moving on to polymeric matrix multi-materials, it’s not unusual to use fused deposition modelling. Transitioning to the year 2004, Le et al. [26] tried to improve the surgical repair of skull fractures or deformities with polycaprolactone-b-tricalcium phosphate-heparan sulphate, and they noticed differences in properties were the increase in osteo-conduction, bioactivity and non-toxicity. Ten years after Tekinalp et al. [27] mixed ABSs with carbon fibres. This allowed for the increase of the flexion resistance and the Young’s modulus.

Another combination is metal with ceramics. It is possible to find a lot of research using the LENS™ and the SLM process. Heer et al. [28] achieved the increase of hardness and the decrease of porosity using AISI 316 plus BN through the LENS™ process. Tian et al. [29] used the SLM process and reach the increase in hardness and the breaking stress.

### 3.2. Materials

Table 2 compiles different combinations of materials that were carried out by different researchers and with different processes.

#### 3.2.1. Polymers and Composites

3D printers utilising polymers have consistently led the way in innovation, primarily due to the simplicity and cost-effectiveness associated with working with polymers compared to metals and ceramics. Whether it entails a mere alteration of colour or the utilisation of two distinct polymers for constructing a single part, systems have begun integrating various material options to propel the advancement of MMAM [60].

#### 3.2.2. Metal–Metal

While there are several AM methods available for metal printing, most of them face constraints in creating single-metal compositions due to machine-specific limitations, such as a single powder-feeding unit. Consequently, addressing this limitation could involve innovating a novel powder-feeding design incorporating multiple sources for materials. Alternatively, the utilisation of pre-mixed powders presents another avenue for the printing of multifunctional components [61].

#### 3.2.3. Metal–Ceramic

Ceramics find applications in various industries such as the chemical industry, machinery, electronics, aerospace, and biomedical engineering due to their unique functional properties. These properties include high mechanical strength and hardness, good thermal and chemical stability, as well as viable thermal, optical, electrical, and magnetic performance. In particular, ceramics have been extensively utilised in conjunction with metals to create multi-material structures through AM technologies [62,63]. The integration of metal-ceramic materials poses a challenge in the fabrication process because of the distinct thermal-chemical properties of each material type [64]. Notably, the difficulty arises from the fact that the melting point of ceramic materials is higher than that of metals [65,66]. However, despite this challenge, ceramics have been successfully incorporated into metal matrices to enhance various properties of the final composite structure. This integration serves to improve thermal properties, wear resistance, hardness, and mechanical strength, resulting in composite materials with superior overall performance [67].

#### 3.2.4. Functionally Graded Materials (FGMs)

**Figure 9:**
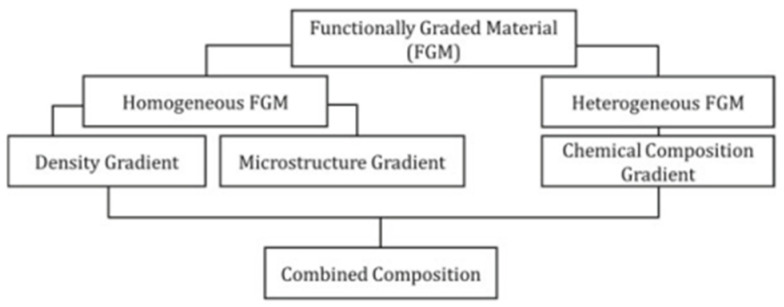
Functionally Graded Materials [71].

FGMs belong to a category of advanced materials distinguished by variations in composition throughout the volume, resulting in corresponding alterations in material properties to meet specific functional requirements [68,69]. Application of FGMs holds significance as it consolidates multiple properties within a single part, eliminating abrupt interfaces among gradient zones and thereby enhancing interfacial strength. The utilisation of FGM can also yield improved material properties and efficiency compared to traditional alloys and metals. FGM parts enable precise control over properties such as weight, modulus of elasticity, fracture toughness, wear resistance, and hardness within a component [70]. Figure 9 shows the classification of FGMs.

**Figure 10:**
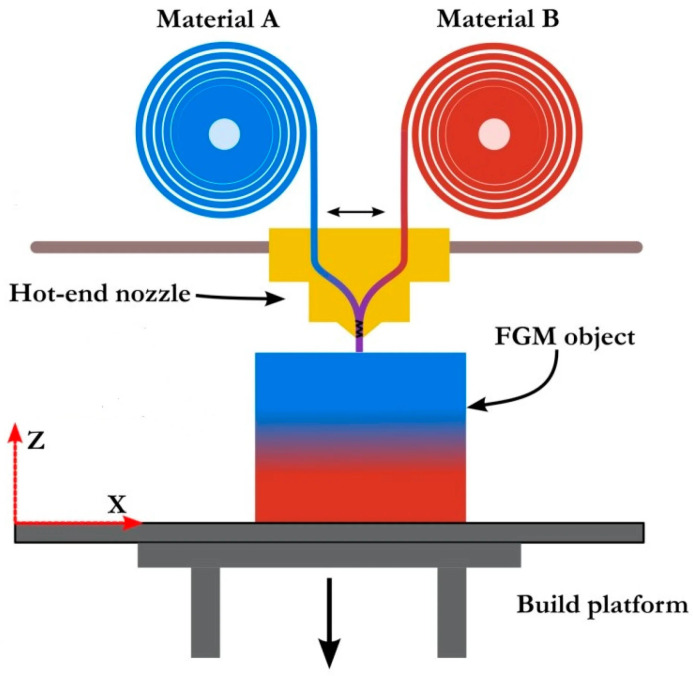
Schematic representation of functionally graded materials fabricated by material extrusion (MEX) [73,74].

Functionally graded MMAM addresses the multi-material aspect by employing a dynamically generated gradient approach or intricate morphology. The geometry and arrangement of materials govern the functions and properties of the final component, intending to enhance the interfacial bonding between dissimilar or incompatible materials. A compositional transition from a dispersed to an interconnected second-phase structure―layered and graded with discrete compositional parameters or smooth concentration gradients―can be achieved to mitigate the adverse properties associated with two dissimilar materials. Consequently, this approach can circumvent the common failures observed in conventional MMAM, such as delamination and cracks induced by surface tension due to discrete variations in material properties [19,72]. All of this is present in Figure 10.

### 3.3. Processes

#### 3.3.1. Material Extrusion

The process material extrusion includes the process FDM, also known as FFF [18]. It can be used with different materials, although plastics and composite materials are preferred. It uses a filament of material that feeds an extrusion head [75]. Inside the nozzle, the material is heated and deposited onto a build plate using a bead-by-bead and layer-by-layer technique [21,76]. Sebbe et al. [30] investigated non-planar FMD by optimising printer hardware and process parameters, demonstrating significant improvements in surface quality and tensile performance, reduced stair-stepping, and support-free fabrication of complex geometries using conventional three-axis FDM systems. Galib et al. [77] reviewed AM of composite materials, highlighting FDM as a cost-effective, versatile extrusion-based technique for thermoplastic and fibre-reinforced composites, while critically discussing process parameters, anisotropy, reinforcement strategies, and limitations affecting mechanical performance. Khondoker et al. [78] did a study to design a static mixer that was placed inside the extrusion head. The object was to mix different polymers. The design tries to improve the adhesion of dissimilar thermoplastics. This concept is illustrated in Figure 10.

There is another example developed by Tian et al. [29] in 2016. The authors want to produce 3D printed continuous carbon fibre reinforced PLA composites. To do this, the extruder head is fed with two distinct materials, where the first filament is PLA, and the second one is carbon. The materials are separated on different spools.

More recently, in 2020, Kennedy and Christ [79] fabricated multi-materials constituted by TPU and PLA. They used a modified Printrbot Simple PRO. They also used a repeater firmware to adjust the mixing ratios that provide material flow. The hot-end heater block was custom and has two inputs and one output. An additional, non-filament input is present to facilitate the introduction of a mixing element, which can traverse the entirety of the melting chamber. The hot-end consists of six components: a custom heater block, two E3D V6 heat breaks with their respective heat sink, the mixing element, the mixing element gasket, and a standard brass nozzle. Integrating a stepper motor for the operation of the mixing element enables independent control of the polymer feed rate and mixing element velocity. Additionally, linking the mixing element stepper motor to the printer’s controller board allows for the modulation of the mixing rate, ensuring proportional mixing speeds relative to the polymer flow rate.

#### 3.3.2. Direct Energy Deposition (DED)

The process of direct energy deposition uses an electric arc, electron beam or laser as a source of energy, and in this process, the metal is melted as it is released by the deposition nozzle [1]. Table 3 contains the different types of DED processes.

#### 3.3.3. LENS™

**Figure 11:**
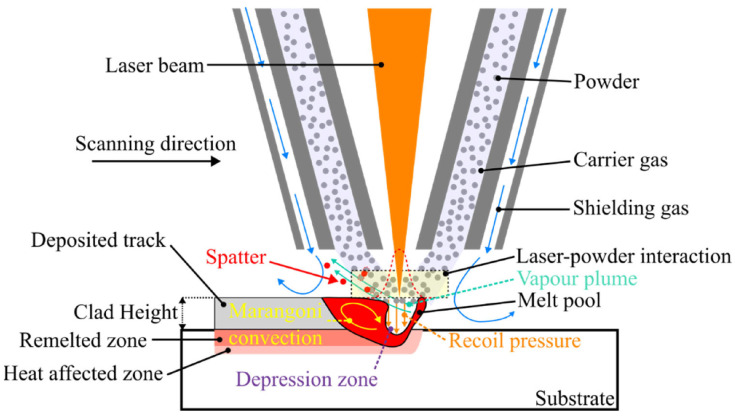
Schematic illustration of melt pool morphology during the LENS™ process. The thermal distribution within the laser beam is assumed to follow a Gaussian profile, represented by the red dashed line [91].

Selective melting of powder occurs using a laser or electron beam, where the scanning or melting pattern consists of pulses or continuous lines. After completing one layer, the table descends, and a roller or scraper evenly spreads a new layer of powder (Figure 11). This process is then repeated to selectively melt the subsequent layer [88]. Researchers have extensively explored the fabrication of multi-material parts through the Directed Energy Deposition process. A recent study by Ke et al. [90] delved into the creation of compositionally graded doped hydroxyapatite coating on titanium. This was achieved using laser and plasma spray deposition techniques to fabricate a bone implant. The investigation presented an innovative approach to improve both the mechanical and antibacterial properties of plasma-sprayed hydroxyapatite coating interfaces. This advancement holds significant potential for applications in load-bearing orthopaedic and dental implants.

Yan et al. [92] did a study to optimise the process parameters of LENS™ using multi-materials like INCONEL^®^ 718 [93,94] and ceramic powders. The cross-sectional view of a modifiable coaxial LENS™ workspace involves a continuous wave laser creating a melt pool on the substrate. Simultaneously, nozzles inject powders into the melt pool within an inert environment, with argon serving as the feeding gas. The cladding layer takes shape as the melt pool materials cool down and solidify. The outcomes indicate that the software developed in-house played a crucial role in minimising energy consumption and material wastage, concurrently enhancing the efficiency of powder melting.

#### 3.3.4. Material Jetting (MJ)

The MJ technology employs printheads for deposition, utilising jets of molten material that, as they are placed on the layer, undergo cooling and solidification. This material jetting process mimics the operation of a standard inkjet printer, where successive layers are added to construct a cohesive object [1]. Several research studies were performed to fabricate multi-material parts using the MJ process. Vu et al. [95] produced acrylic photopolymers through the MJ process to investigate the fracture characteristics of material interfaces. Specifically, elastomeric (TangoBlackPlus) and acrylic (VeroWhitePlus) materials were utilised to create fracture specimens. The findings revealed that failures predominantly occurred at the zones of material interfaces. Additionally, the results demonstrate that gradient material interfaces could be effectively achieved in this process, ultimately improving the joint strength between the two material components.

#### 3.3.5. Vat–Photopolimerization

**Figure 12:**
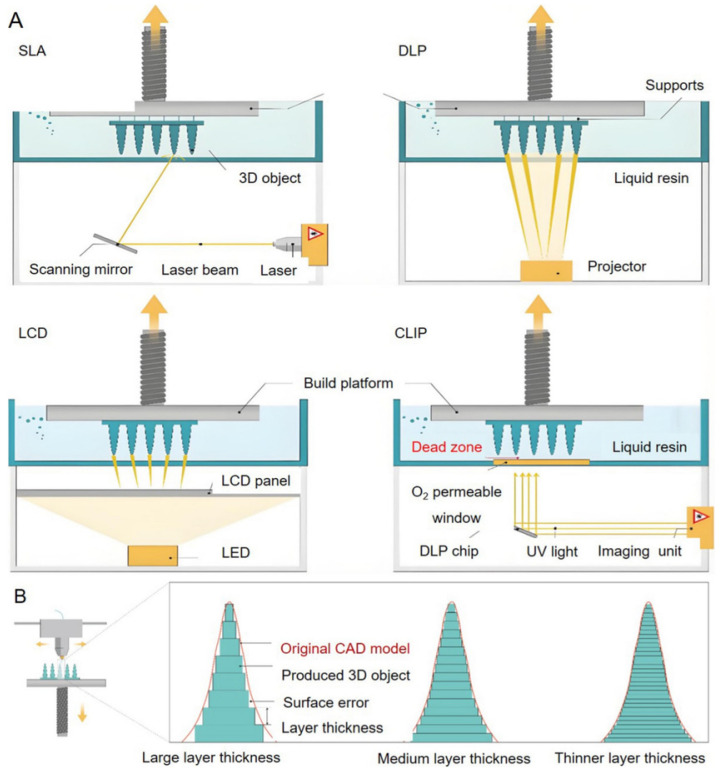
Scheme of (**A**) The working principle of SLA, DLP, LCD, and CLIP (**B**) Staircase phenomenon for different layer thicknesses [100].

The process of photopolymerization includes the processes of SLA, DLP, and Continuous Digital Light Processing (CDLP) [96]. The process SLA uses photopolymer resin to fabricate materials layer by layer [97]. Utilising multiple materials in the VAT process poses challenges in effectively managing contamination between them. The liquid feedstock material is typically placed in a vat, and two different technological processes can be employed: either top-down or bottom-up [98,99]; as illustrated in Figure 12.

Nonetheless, there has been a recent proposal to address this issue by introducing automation to the technique, allowing for the fabrication of multi-material parts by Choi et al. [101] and Shaukat et al. [102]. The rotary vat carousel system consists of four circumferentially positioned stainless steel vats (203.2 × 228.6 mm^2^). The MMSL machine has a maximum build envelope of 165.1 × 120.7 mm^2^, with a vertical build height limited to 120.7 mm due to physical constraints. The cross-section of the MMSL machine’s vat is constrained by the maximum torque output (425 Nm) of the ADRT-200 direct drive rotary stage, resulting in MMSL resin vats with a volume of 9 litres, compared to the original 250/50 vats’ 32.21 litres. Vat rotation is facilitated by a 50.8 mm diameter aluminium shaft attached to the high-precision direct drive rotary stage, chosen for its accuracy (±30 arc-s), substantial torque output, and superior angular positioning, velocity control, and load capacities (30–173 kg). The vat carousel frame features a central 6061 aluminium plate (26.0 mm thick) with aluminium support columns (25.4 mm × 25.4 mm cross-section) designed to support each resin-filled vat, each incorporating a small rectangular (53 mm × 25 mm) confinement section housing a floating target for level sensing.

The utilisation of this machine was applied in the fabrication of a rook or castle, a frequently employed structure. This process involved employing two intricate models, each comprising three sub-models, and utilising three distinct resins. In the construction process, each build involved the fabrication of a sub-part either on the platform or directly onto a previously constructed section. The final parts were successfully realised from these designs.

#### 3.3.6. Direct Ink Writing (DIW)

**Figure 13:**
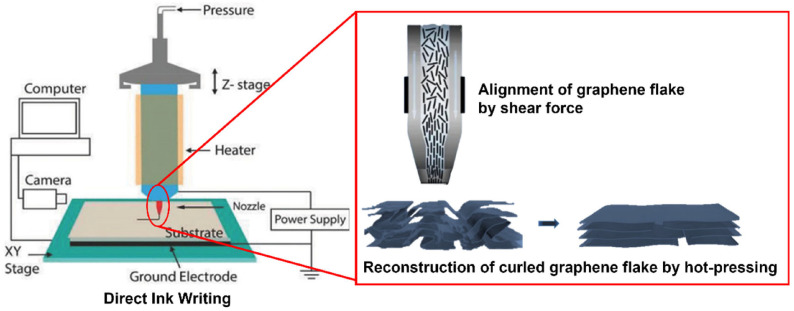
Schematic illustration of graphene flake alignment induced by DIW and subsequent hot pressing [105].

The term “direct ink writing” describes fabrication methods that employ a computer-controlled translation stage, which moves a pattern-generating device, that is, an ink-deposition nozzle, to create materials with controlled architecture and composition. Several direct ink writing techniques have been introduced that can pattern materials in three dimensions. They can be divided into filamentary-based approaches, such as robocasting (or robotic deposition micropen writing, fused deposition, and droplet-based approaches, such as ink-jet printing and hot-melt printing [103,104]. This process is very similar to the FFF, although it uses a heating source to produce the components (Figure 13) [18].

García Rocha et al. [106] presented methodologies for the direct ink writing of diverse materials with elevated spatial resolution that rely on precision motion control systems. This involved employing an XYZ gantry robot with an accuracy and repeatability of 1–3 μm, in all three directions. Several enhancements and modifications to the basic setup mentioned earlier broaden the technique’s versatility and expand the range of formulations, thereby increasing the complexity of the printed object [107]. Examples include the incorporation of active mixers and droplet generators, attaching CCD cameras or UV-light sources for polymer curing, manipulating humidifiers, suspension baths for embedded printing, and employing heated syringes and/or platforms.

Skylar-Scott et al. [108], incorporated a mixer into the extrusion head to enhance the material mixing process, leading to improved homogeneity and uniformity in material blends. This modification enables the operator to swiftly adjust the material blending ratio during the deposition process. Also Tan et al. [109] and García Rocha et al. [106] performed studies on the DIW AM.

This method integrates ultrarapid multi-material switching to enhance complexity, coupled with multi-nozzle printing to boost build speed. Through MM3D printing, the researchers successfully generated intricate architectures consisting of multiple materials. This approach allowed for precise control over the composition, function, and structure of the printed objects in a voxel-by-voxel manner [108].

Pelz et al. [59] developed a custom DIW system to facilitate the printing of carbide components with varied structures. Aqueous inks containing boron carbide and silicon carbide powders were employed as feedstock for printing heterogeneous carbide specimens. The custom DIW system underwent evaluation to determine its capability to print and mix multiple materials at predefined ratios. Subsequently, the printed specimens underwent testing to assess shape retention, density, microstructure, and mechanical properties. The feed system comprises two units, each independently controlled to dispense two distinct ceramic inks to the print head. Each unit employs a leadscrew-driven plunger for precise control over the volumetric feed rate. The ceramic ink is conveyed through 4 mm inner-diameter Teflon tubes to the print head, housing an auger for in-line mixing and accurate extrusion.

#### 3.3.7. Selective Laser Melting (SLM)

**Figure 14:**
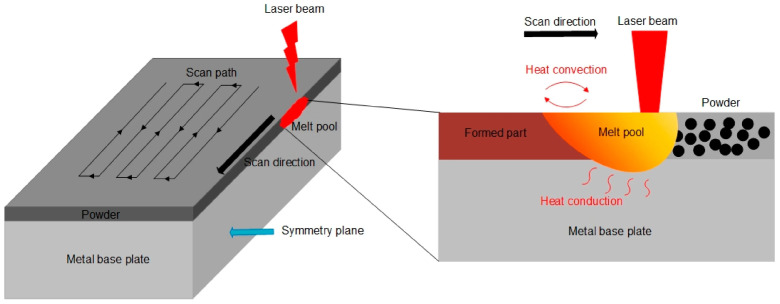
SLM process scheme [110].

The SLM process uses one or more laser beams to promote the fusion of specific regions within the powder bed. A fibre laser is used in the equipment. The equipment commonly features galvanometric mirrors that direct the beam along the available area, influencing the build rates of this technology [1]. Figure 14 presents a scheme of the process.

Liu et al. [111] used the SLM250HL equipment to do their multi-material consisting of gas atomised 316L stainless steel and C18400 alloy powders. The authors obtained a macroscopic view of the samples that showed that the copper/steel interface was intact.

Koopmann et al. [112] initially used X38CrMoV5-3 and a ceramic powder that was obtained by dry mixing ZrO_2_ and Al_2_O_3_. Microstructural analysis unveiled the development of essentially four distinct microstructures, each exhibiting a unique appearance, along a singular melting line of the ceramic material.

#### 3.3.8. Hybrid Additive Manufacturing (HAM)

HAM refers to integrating two or more distinct processes and machines. The fundamental objective of this approach is to address the constraints of MMAM techniques and enhance both quality and productivity. The primary objective of employing this method is to address the limitations of MMAM techniques to enhance both quality and productivity [113,114]. Lately, DMG MORI’s Lasertec 125 3D hybrid system was released, which is a HAM process that creates, maintains and repairs workpieces up to 1250 mm wide and 750 mm high, weighing up to 2000 kg. This system uses a five-axis material fabrication and five-axis milling process within the same machine. It is noteworthy that automatic shifting between laser deposition welding and simultaneously five-axis milling in a single manufacturing process reduces fabrication time by up to 80% [114].

#### 3.3.9. Functionally Graded Additive Manufacturing (FGAM)

The FGAM is conducted by the ISO/ASTM TR 52912:2020(E) standard [115], and it says that heterogeneous compositions with multiple materials are achievable using conventional 3D printers with multiple nozzles to deliver different materials to the platform. Multi-material (MM) FGAM aims to enhance the interfacial bond by eliminating the distinct boundaries between dissimilar or incompatible materials. This will significantly reduce mechanical stress concentrations and thermal stress caused by different expansion coefficients [115,116].

It is a manufacturing process conducted layer by layer, wherein the arrangement of materials within a component is systematically altered to achieve a specific intended function [72], in other words involves a gradual variation of the material organisation to reach a function. Table 4 shows different processing methods of FGAM.

### 3.4. Main Parameters

During the project it is important to consider the different variables, such as global parameters of AM [1]: (1) 3D printing layer height; (2) Line width; (3) Wall thickness; (4) Internal surface infill; (5) Support structure setup and generation; (6) Build platform adhesion settings; (7) Printing material setup; (8) Extrusion speed configuration. Beyond these parameters, it is possible to separate different parameters due to the different processes. This is schematized in Table 5.

### 3.5. Fundamental Mechanisms Governing Interface Behaviour in MMAM

The performance and reliability of multi-material additively manufactured components are fundamentally governed by complex thermophysical and metallurgical phenomena occurring at material interfaces during deposition and solidification. Unlike conventional joining processes, MMAM involves repeated rapid heating and cooling cycles, producing highly non-equilibrium microstructures and steep thermal gradients that strongly influence interfacial bonding and defect formation.

#### 3.5.1. Diffusion and Interfacial Mixing

During multi-material deposition, atomic diffusion across material interfaces plays a critical role in bonding formation. Elevated local temperatures generated by laser or extrusion-based processes promote interdiffusion between adjacent materials, resulting in transition zones rather than perfectly discrete interfaces. For instance ZainElabdeen et al. [118] conducted a comprehensive review of hybrid LPBF fabrication of similar and dissimilar metals, examining interfacial diffusion behaviour, metallurgical bonding mechanisms, intermetallic formation, residual stress development, and process–structure–property relationships. The extent of diffusion depends on temperature exposure time, material compatibility, and diffusion coefficients of the interacting elements. In Leubecher et al. [119] study, a systematic investigation of in-situ substrate and extrudate temperature modulation was performed, in LS-MEX of PA6–40 wt% CF, correlating tensile performance, fracture morphology and WAXD-derived crystalline orientation with controlled thermal boundary conditions. Insufficient diffusion may lead to weak metallurgical bonding, whereas excessive intermixing can generate undesirable phases or compositional instability. Wang et al. [120] steered a comprehensive review of molecular dynamics investigations into bimetallic interfacial behaviour, analysing atomic diffusion, intermetallic formation, deformation mechanisms, and machine-learning-enhanced potentials for predicting strength and stability.

#### 3.5.2. Phase Formation and Intermetallic Stability

In metal–metal and metal–ceramic systems, interfacial reactions are frequently governed by phase equilibria described by binary or ternary phase diagrams. Samad et al. [121] exhibited a comprehensive review of bimetallic MMAM systems, examining alloy compatibility, interfacial microstructure evolution, brittle intermetallic formation, thermal mismatch effects, and process–structure–property relationships governing mechanical performance. Rapid solidification conditions typical of AM processes may promote the formation of metastable or brittle intermetallic compounds, particularly when elements exhibit limited mutual solubility. Verma et al. [122] reviewed multiplicity in MMAM, analysing process adaptations (DED, PBF, extrusion, jetting), material combinations, interface challenges, graded structures, scale effects, and capability-driven applications across polymeric, metallic, ceramic, and hybrid systems. These intermetallic phases often possess high hardness but low fracture toughness, increasing susceptibility to crack initiation and propagation under thermal or mechanical loading. Functionally graded material strategies mitigate this effect by gradually modifying composition and reducing abrupt thermodynamic incompatibilities.

#### 3.5.3. Solidification Behaviour in Graded Regions

Solidification dynamics within graded material regions differ significantly from single-material AM builds. Vanaei [123] provided a comprehensive overview of MMAM of metals and alloys, examining process principles (DMLS, SLM, EBM, PBF), material selection strategies, interface challenges, gradient design methodologies, and sector-specific industrial applications. Variations in composition alter local melting temperatures, nucleation kinetics, and dendritic growth behaviour. Schneck et al. [124] demonstrated fabrication of a steel–copper functionally graded injection nozzle via PBF-LB/M, analysing transition-zone porosity (≈98.8% density), vertical cracking, cross-contamination, and process-dependent defect mechanisms in industrially relevant geometries. Rapid cooling rates, often exceeding 10^3^–10^6^ K/s in laser-based systems, refine microstructures but may also introduce segregation, residual porosity, or anisotropic grain growth aligned with thermal gradients. Bandyopadhyay and Heer [60] conducted a comprehensive review of multi-material AM across polymers, metals, and metal–ceramic systems, analysing processing routes, interfacial bonding mechanisms, gradient strategies, microstructural evolution, and emerging multifunctional applications. Controlled compositional transitions can stabilise solidification fronts and reduce defect formation across interfaces.

#### 3.5.4. Microstructural Evolution Under Rapid Thermal Cycling

Repeated thermal cycling inherent to layer-by-layer fabrication leads to continuous microstructural evolution, including grain coarsening, phase transformation, and residual stress accumulation. Denlinger et al. [125] developed a three-dimensional thermoelastoplastic finite element framework for large-scale electron beam deposition, quantifying distortion accumulation, repeated thermal cycling effects, residual stress development, and layer-wise heat input under industrially relevant conditions. Differences in thermal expansion coefficients between materials generate localised strain fields during cooling, contributing to delamination or interfacial cracking. These effects are particularly pronounced in metal–ceramic and polymer–metal combinations due to large thermomechanical mismatches. Ma et al. [126] investigated SS 316L/IN718 functionally graded materials fabricated by LDED, combining multiphase fluid modelling and thermo-mechanical simulations to elucidate non-equilibrium solidification, solute segregation, residual stress accumulation, and crack initiation mechanisms.

#### 3.5.5. Residual Stress Development and Modelling Approaches

Residual stresses arise primarily from non-uniform thermal contraction and constrained solidification during deposition. Numerical modelling approaches, including finite element thermal–mechanical simulations and phase-field modelling, are increasingly employed to predict stress evolution and optimise deposition strategies. Yang et al. [127] developed a coupled thermal–fluid and phase-field model to simulate grain evolution in TiB_2_-reinforced 316L during SLM, demonstrating particle-induced heterogeneous nucleation, Zener pinning, grain refinement, and suppression of columnar growth and coarsening. Process parameter control, graded transitions, and preheating strategies have been demonstrated as effective approaches for mitigating stress accumulation and improving interfacial stability.

#### 3.5.6. Nanoscale Characterisation of Multi-Material Interfaces

Understanding interfacial behaviour in MMAM increasingly relies on nanoscale characterisation techniques capable of resolving compositional gradients, diffusion zones, and phase transformations occurring across material transitions. Conventional optical and scanning electron microscopy provide valuable morphological information; however, advanced techniques are required to investigate nanoscale bonding mechanisms and defect formation.

Transmission electron microscopy (TEM) and high-resolution scanning transmission electron microscopy (STEM) enable direct observation of intermetallic phase formation, dislocation structures, and diffusion layers at material interfaces. Energy-dispersive X-ray spectroscopy (EDS) and electron energy loss spectroscopy (EELS) mapping are commonly employed to quantify elemental gradients within graded regions.

Atom probe tomography (APT) has recently emerged as a powerful technique for analysing atomic-scale compositional variations in functionally graded materials, allowing three-dimensional reconstruction of diffusion behaviour and segregation phenomena. Additionally, synchrotron-based X-ray diffraction and nano-indentation methods have been applied to evaluate local mechanical properties and residual stress distributions across interfaces.

The integration of nanoscale characterisation with process modelling is expected to play a key role in improving predictive understanding of interface stability and long-term reliability in MMAM systems.

#### 3.5.7. Predominant Degradation Mechanisms in MMAM Interfaces

The structural integrity and functional performance of multi-material additively manufactured components are influenced by several interrelated degradation mechanisms that may be categorised as interfacial, microstructural, thermal, and mechanical in nature. Interfacial degradation primarily arises from insufficient diffusion bonding, brittle intermetallic formation, chemical incompatibility, and compositional instability at material transitions. Microstructural degradation mechanisms include segregation, porosity, anisotropic grain growth, grain coarsening under cyclic reheating, and metastable phase formation induced by rapid solidification.

Thermal degradation is largely governed by thermal expansion mismatch, steep thermal gradients, and cyclic heating effects, which promote residual stress accumulation and interfacial delamination. Mechanical degradation manifests through crack initiation at brittle intermetallic layers, fatigue-driven crack propagation under cyclic loading, creep deformation in graded regions, and stress concentration at abrupt compositional transitions. These mechanisms are strongly coupled and process-dependent, necessitating integrated design strategies incorporating graded transitions, process optimisation, and predictive thermo-mechanical modelling to ensure long-term reliability.

### 3.6. Equipments

In this chapter, it’s shown some 3D printing machines that are specialized to use multi material. The first example is the Exam 255 3D printer AIM3D [128]. This equipment offers a solution that can be used across materials like metal, plastic or even ceramic. The systems of the CEM process achieve tensile strengths that come close to classic thermoplastic, mould-bound injection moulding. Companies like Brose, Schunk, Schaefler and BASF are customers of this product.

The company Schaeffler has invested in a multi-material AM machine, which is expected to be available from 2024. In the works of Oel et al. [129] and Clare et al. [130], the Aerosint’s Selective Powder Deposition (SPD) technology is used. It is a multi-material recoater for multi-material LPBF, which is the core enabler of Schaeffler OmniFusion 3D, which is appropriately suitable for metals and ceramic materials.

In 2019, the world’s largest multi-material AM system, called KRAKEN, which is an EU-funded project that created an automated, robotic machine for hybrid multi-material manufacturing combining both subtractive and novel additive technologies [131,132]. Capable of producing parts of up to 20-metrelong with a 0.1 mm precision, the KRAKEN machine has automatically interchangeable additive and subtractive heads, the capacity to achieve high quality and accurate results, including internal and difficult-to-reach areas, and effective control over thermal stress and deformation [133]. There are already results from this project, like a new polyurethane paste, a new resin extrusion process, wire arc AM and a metallisation process [134].

Furthermore, a hybrid manufacturing machine for large component production was also built. The maximum working area is 2000 × 800 × 600 cm^3^.

The Aerosint company has a large catalogue of equipment and produced parts. They specialised in multi-material LPBF and multi-material sintering [135]. Starting with STD technology, this is applied to techniques like LPBF, Binder Jetting or Die filling & Sintering [136]. In this moment, they have two machines of this kind, the AconityMIDI+ printer and the AconityMICRO.

### 3.7. Applications

#### 3.7.1. Biomedical Engineering

Numerous biomedical applications have been developed using MMAM, with a particular focus on creating 3D engineered tissue constructs that emulate biological systems in tissue engineering. Given the structural complexity of natural tissues, which comprise multiple cell types, MMAM is indispensable in the fabrication of engineered tissue constructs. A recent achievement involves the 3D printing of neuronal and glial progenitor cells to construct a 3D spinal cord tissue-like structure [137]. In addition to tissue engineering, MMAM has been instrumental in crafting various biomedical devices such as microneedle arrays and diagnostic devices [138,139]. Transdermal microneedle arrays typically consist of two distinct parts, namely microneedle bodies and a substrate, serving different functions such as drug delivery, biofluid collection, structural support, and skin protection. Employing two materials with diverse properties enhances the performance of microneedle arrays. Similarly, diagnostic microfluidic devices designed for measuring pharmaceuticals in biological fluids feature multiple functional parts, including an optically transparent main body for visual detection, electrodes for electrokinetic transport, and membranes for screening target molecules―all fabricated using MMAM [140,141].

#### 3.7.2. Soft Robots

Soft robots are characterised by their compliant and flexible bodies, allowing for intricate actuations and motions that would be challenging for conventional robots with rigid bodies and joints [142]. The continuous evolution of AM and the growing range of printable materials have led to a shift toward the direct manufacturing of soft robots using Microscale AM (MAM) [143]. This approach simplifies manufacturing procedures while maintaining functional complexity. One notable example involves pneumatically driven elastomeric actuators, featuring an elastomeric body with a surface adorned by a stiff reinforcing material aligned at a specified orientation through DIW [144]. The orientation of the reinforcement dictates the local anisotropic deformation during inflation, resulting in diverse motions such as elongation, contraction, and twisting. DIW-driven programming of reinforcement was also employed to control the complex swelling of a hydrogel for 4D printing [145]. MMAM enables the direct integration of various functional components essential for actuation. For instance, a hybrid MMAM approach combining DIW and MJ was utilised to print a silver-nanoparticle ink as a resistive heating element on a stimuli-responsive liquid crystal elastomer (LCE) that exhibits shape change with temperature for reversible soft robotic actuation. This hybrid approach was also employed to directly print a stiffness-tunable soft actuator comprising a soft elastomeric body connected with a pneumatic system, a shape memory polymer (SMP) layer for stiffness control, and silver nanoparticle ink for resistive heating [146].

#### 3.7.3. Eletronics

MAM plays a crucial role in the direct manufacturing of 3D electronic devices, allowing for the integration of electrically dissimilar materials such as conductors, semiconductors, and dielectrics [147]. The addition of the third dimension enables the further miniaturisation of 3D electronics, resulting in a smaller footprint and form factor. An illustrative example is the 3D printing of a magnetic sensor with integrated electronic components and conductive paths, showcasing improved volume utilisation by leveraging the design in all three dimensions [148]. A recent advancement includes the demonstration of a highly stretchable electronic LED board achieved by printing a stretchable conductive hydrogel on an elastomer [149]. The hydrogel’s stretchability, reaching up to 1300%, ensures that the electric circuit maintains conductivity even under significant deformation. The high design and manufacturing flexibility offered by AM also pave the way for the exploration of new designs in energy storage devices for higher capacity. A fully 3D printed and packaged Li-ion battery has demonstrated high areal capacity [150]. Additionally, interconnected photodetector arrays, printed on both planar and hemispherical surfaces, exhibited high sensitivity and a wide field-of-view. This was achieved by concurrently printing five different materials, including a photoactive layer, a transparent anode, a silver nanoparticle interconnect, an electrical insulating layer, and a cathode, on substrates with arbitrary geometries using MMAM.

#### 3.7.4. Aerospace Industry

Within the aerospace industry, Material and Manufacturing for Advanced Manufacturing is employed to achieve intricate shapes and processing techniques that are unattainable through conventional methods. Components such as electric motors and turbine engines, which incorporate sub-elements made of different materials and structures, are examples of parts constructed with the assistance of Material and Manufacturing for Advanced Manufacturing to attain unique and superior properties, including microstructure, mechanical strength, electrical conductivity, thermal resistance, and magnetic characteristics [151].

LBPF stands out as one of the AM processes suitable for building metallic and non-metallic materials. This method can be utilised independently or in combination with other AM processes to fabricate components with high geometrical resolution. These components may comprise various material combinations, such as metal–metal, metal–ceramic, and metal–polymer, showcasing the versatility and advanced capabilities of MMAM in the aerospace sector [152].

### 3.8. Critical Issues

One of the biggest problems of MMAM is the fact that when we are trying to join different materials, they have thermos-mechanical properties, such as the fusion temperature. This can be a problem because the temperatures are two different, and one of the materials may become degraded. Some challenges are specific to each MAM method, and some are common. The shared difficulties stem from the unconventional practice of constructing a monolithic component using various materials. The amalgamation and fusion of diverse materials within a single component pose the potential for the formation of intermetallic phases and interfaces, introducing the risk of undesirable material behaviours. This necessitates a case-by-case examination for each combination and level of mixing [153]. Thermal expansion mismatch between dissimilar materials represents one of the primary sources of interfacial stress in MMAM components. Typical coefficients of thermal expansion (CTE) vary significantly across material classes, for example, polymers (50–150 × 10^−6^ K^−1^), metals (10–25 × 10^−6^ K^−1^), and ceramics (3–10 × 10^−6^ K^−1^). During cooling from processing temperatures, these differences generate thermally induced stresses that may exceed interfacial bonding strength, promoting delamination or crack initiation. Functionally graded transitions reduce these stresses by progressively distributing strain across compositional gradients.

In the context of LPBF, incorporating multiple materials becomes an intricate affair that impacts the entire process chain. The complexities arise in tasks such as segregating individual material powders, reclaiming them, and recycling the multi-material component, which continue to present challenges [152].

In DED, specific challenges arise, including low resolution in terms of material mixing locations, leading to substantial post-processing requirements. Moreover, when blown powder is employed, a significant portion of the material utilised does not contribute to the final part, being either lost or contaminated and not reused. This situation diminishes the overall efficiency of the method [153,154].

Although there is a growing presence of multi-material AM systems facilitating the creation of functionally graded materials in both polymer and metal contexts, the widespread adoption of these systems is restricted due to uncertain behaviour at material interfaces and a lack of support from design software. To be more specific, currently available commercially-based software packages do not provide designers with the capability to easily model or analyse geometries involving multiple materials and their associated anisotropies [155,156,157].

Addressing issues of material system contamination during transitions to different materials is a significant concern in the majority of MMAM systems. This introduces imperfections in the processes and poses challenges to the effective reuse of materials.

The challenge of bonding dissimilar materials has persistently existed, even within traditional manufacturing methods. Similarly, ensuring a robust bond between dissimilar layers in MMAM is a critical aspect that requires careful consideration. Currently, most AM systems utilise STL files as standard input, representing a surface approximation without information about material content. There is a growing need for a robust Computer-Aided Design (CAD) system designed for multiple material pre-processing. This system would empower operators to identify material types in different regions within a part. Utilising multiple materials in a single process can lead to interruptions and time loss in certain MMAM or hybrid processes due to material/component changeovers during the build process. The challenge lies in ensuring that the application of multiple materials or additional components minimally disrupts the overall process. Hybrid and multi-axis systems: While some AM processes may not inherently excel in multiple materials processing, they do demonstrate a strong capability for integration with other AM or conventional techniques, leading to the creation of efficient hybrid systems. For instance, dry powder printing can be seamlessly integrated with a laser sintering process to produce multi-material outcomes. Additionally, processes like LENS, FDM [30,77], and LOM have all exhibited advantages from the added complexity of motion. The challenge lies in effectively integrating different processes to achieve a harmonised system. Beyond the expansion of blended composites, there is a likelihood of adopting various other forms of multiple material systems. This prospect could be the most intriguing advancement, particularly as electrically or thermally conductive materials, semiconductors, liquid crystals, carbon nanotubes, functional ceramics, and more become increasingly utilised. Moreover, alongside the continued progress in embedded technologies, it is conceivable to create sensors directly from their fundamental material components within the part itself using direct write techniques [12].

### 3.9. Quantitative Comparison of Materials and Processes in MMAM

While previous subsections describe material systems and processing approaches employed in MMAM, a comparative evaluation of reported quantitative performance indicators provides additional insight into technological capabilities and maturity levels. Based on the analysed literature, key performance metrics frequently reported include interfacial bonding strength, thermal conductivity, residual stress behaviour, mechanical performance, and process stability. Table 6 summarises representative quantitative ranges reported across major material systems and AM processes.

FGM strategies consistently demonstrate improved stress redistribution and reduced interfacial failure compared with discrete material transitions, highlighting their increasing relevance for next-generation MMAM systems.

## 4. Discussion

**Figure 15:**
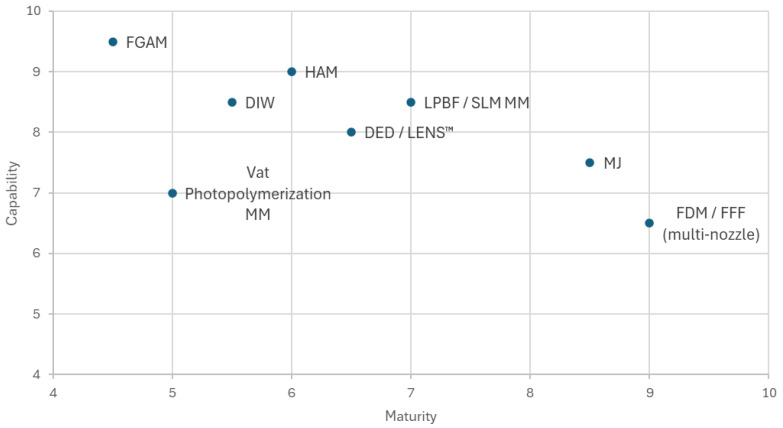
Technology maturity versus multi-material performance capability of major AM processes used in MMAM, based on synthesis of reported industrial adoption, process stability, material compatibility, and functional integration potential across the reviewed literature.

Beyond individual process characteristics, evaluating the relative technological maturity of MMAM approaches provides insight into current industrial readiness and future development trajectories. Figure 15 presents a qualitative maturity–capability mapping derived from the analysed literature, considering process stability, scalability, material compatibility, and demonstrated application deployment. Extrusion-based systems and material jetting technologies currently exhibit the highest industrial maturity, whereas functionally graded and hybrid manufacturing platforms represent rapidly evolving research directions with significant future potential.

The discussion of the results presented in this study reveals significant insights into MMAM. Analysing the data and interpreting the findings, the importance of specific elements in the process and how these results contribute to the current understanding in Engineering are highlighted. In the specific context of recurring patterns, there is a notable inclination towards innovation, both in the combination of materials and in adapting equipment and different methods to achieve good compatibility between materials, even with differences in their thermochemical properties. These results corroborate findings from relevant references in Engineering and strengthen the validity of the conclusions presented. In summary, the critical analysis of the results and the reflection on the produced articles indicate general conclusions about MMAM. Confidence lies in the substantial contribution of this work to the advancement of knowledge in Engineering, and it is hoped that it will serve as a foundation for future research, providing a lasting impact on the academic community.

The challenges identified across the reviewed studies are strongly linked to underlying thermodynamic and microstructural mechanisms occurring during multi-material deposition. Interface stability, residual stress formation, and defect evolution are governed by coupled diffusion, phase transformation, and rapid solidification phenomena, highlighting the need for integrated materials–process modelling approaches in future MMAM development. Long-term reliability remains a critical challenge for industrial deployment of MMAM. Repeated thermal and mechanical loading may induce thermal fatigue due to cyclic expansion mismatch between materials, leading to progressive crack propagation at interfaces. These effects are particularly relevant in aerospace and electronic applications operating under fluctuating temperature conditions.

In electrically functional MMAM systems, electromigration phenomena may also arise when conductive pathways are integrated within polymer or ceramic matrices. Atomic diffusion driven by current density can gradually degrade conductive tracks, reducing device lifetime. Recent studies indicate that reliability prediction increasingly relies on coupled thermo-mechanical simulations combined with accelerated fatigue testing and microstructural monitoring. Future research directions, therefore, emphasise durability assessment, lifetime modelling, and standardised qualification methodologies for multi-material AM components.

## 5. Main Contributions of Work

Rooted in a critical review of references, this study delves into the realm of MMAM. It encapsulates recent research on cutting-edge innovations in both machines and materials, providing an extensive compilation of definitions. Furthermore, it navigates through the works of distinguished researchers in the field, shedding light not only on noteworthy discoveries but also on unprecedented alterations to equipment and inventive combinations of materials. By synthesising a diverse array of studies, this review aspires to present a comprehensive perspective, capturing the latest findings and enriching our understanding of MMAM. Importantly, this exploration seeks to contribute to the wider scientific community by offering insights that may inspire and inform further advancements in this dynamic field.

## 6. Limitations of the Study

The present study is not without its limitations, and it is crucial to acknowledge these constraints for a nuanced interpretation of the findings. Firstly, the scope of the literature review was confined to references spanning the years 1998 to 2023. While this timeframe provided a substantial foundation, it is essential to recognise that developments or key insights outside this period may not be fully captured. Future research may consider extending the temporal scope to ensure a more comprehensive understanding of the evolution of the subject. Secondly, the temporal constraints imposed by a relatively short timeframe for conducting the study, less than three months, inherently limit the depth and breadth of the analysis. The condensed schedule may have influenced the thoroughness of the literature review, potentially overlooking certain nuanced aspects or recent contributions. A more extended timeframe would have allowed for a more exhaustive exploration of the literature, enhancing the comprehensiveness of the study. Additionally, it is important to acknowledge a linguistic limitation. The study primarily relied on information available in English, which may have introduced a language bias. This decision inadvertently led to a reduction in the diversity of articles and websites included in the review. Including literature in other languages could have provided a more global perspective and potentially uncovered additional relevant insights. Future endeavours should strive to overcome language barriers to ensure a more inclusive and diverse literature review.

## 7. Conclusions

This systematic review has provided a comprehensive and critically structured synthesis of multi-material additive manufacturing (MMAM), integrating technological developments, materials science mechanisms, process innovations, equipment architectures, and application domains within a unified analytical framework. Guided by a PRISMA 2020-aligned methodology and structured Research Questions (RQ1–RQ5), the study consolidates dispersed literature across polymeric, metallic, ceramic, and hybrid systems to elucidate the scientific foundations, technological maturity, and industrial implications of MMAM.

1. Technological Evolution and Process Integration (Section 3.1, Section 3.2, Section 3.3 and Section 3.4)

The evolution of MMAM has been driven primarily by advances in process integration and machine architecture. Multi-feed directed energy deposition (DED), multi-nozzle material extrusion, selective powder deposition recoating systems, and hybrid additive–subtractive manufacturing platforms have expanded the capacity to fabricate components comprising both discrete and functionally graded material combinations. Functionally graded additive manufacturing (FGAM) has emerged as a pivotal strategy for mitigating abrupt interfacial discontinuities and improving thermomechanical compatibility.

Across major process classes―material extrusion, powder bed fusion (PBF), directed energy deposition, material jetting, vat photopolymerisation, and direct ink writing―distinct performance characteristics are observed. Laser-based systems typically generate rapid cooling rates in the order of 10^3^–10^6^ K·s^−1^, enabling refined microstructures and relative densities frequently exceeding 98–99% under optimised conditions. Extrusion-based and DIW systems offer superior flexibility in polymer–polymer and composite architectures, whereas LPBF and DED demonstrate enhanced metallurgical bonding in metal–metal and metal–ceramic systems. Hybrid manufacturing platforms further improve geometric fidelity and dimensional control, with reported reductions in post-processing time of up to 60–80% in integrated laser–milling systems.

2. Fundamental Interfacial and Microstructural Mechanisms (Section 3.5; RQ1–RQ2)

The structural integrity and functional performance of MMAM components are governed by coupled interfacial, microstructural, thermal, and mechanical phenomena. Diffusion-driven interfacial mixing, phase formation controlled by binary and ternary equilibria, rapid solidification behaviour in graded regions, and residual stress evolution collectively define interface stability.

Rapid solidification, particularly in laser-based systems, promotes microstructural refinement but may also induce segregation and porosity. Reported transition-zone densities in graded PBF systems typically range between 97–99%, although localised cracking remains prevalent in systems with limited mutual solubility. Brittle intermetallic formation in dissimilar metal systems often increases hardness but reduces fracture toughness, thereby elevating crack initiation risk under cyclic loading.

Thermal expansion mismatch represents a principal degradation mechanism. Typical coefficients of thermal expansion (CTE) vary significantly across material classes, with polymers ranging approximately from 50–150 × 10^−6^ K^−1^, metals from 10–25 × 10^−6^ K^−1^, and ceramics from 3–10 × 10^−6^ K^−1^. Cooling from processing temperatures therefore generates substantial thermally induced stresses, which in extreme cases may exceed local interfacial bonding strength and promote delamination.

Functionally graded transitions consistently outperform discrete interfaces, with several studies reporting interfacial strength improvements in the range of 15–40% depending on material pairing and gradient design. Graded architectures reduce stress concentration factors, stabilise solidification fronts, and distribute strain progressively across compositional gradients. Numerical modelling frameworks, including thermo-mechanical finite element simulations and phase-field modelling, increasingly provide predictive capability for stress magnitudes and microstructural evolution. Complementary nanoscale characterisation techniques—such as transmission electron microscopy and atom probe tomography—validate diffusion layer thicknesses, compositional gradients, and phase stability at sub-micrometre scales.

3. Equipment Innovation and Industrial Platforms (Section 3.6; RQ3)

Machine-level innovation has emerged as a critical enabler of MMAM scalability. Multi-material recoating systems, controlled-atmosphere deposition chambers, hybrid laser–milling platforms, and dynamic mixing extruders demonstrate the convergence of materials engineering and system integration. Large-format hybrid systems capable of metre-scale fabrication highlight the transition from laboratory-scale experimentation towards industrial deployment.

However, technological bottlenecks persist. Powder segregation control, automated material switching reliability, and contamination mitigation remain unresolved in many LPBF and DED systems. In blown-powder DED processes, material utilisation inefficiencies and cross-contamination reduce overall process efficiency, occasionally resulting in effective material losses exceeding 20–30% during multi-material transitions.

4. Application-Driven Integration (Section 3.7; RQ2–RQ4)

The expanding application landscape underscores the transformative potential of MMAM. In biomedical engineering, graded metallic implants demonstrate improved stiffness matching relative to cortical bone, mitigating stress shielding effects. In soft robotics, voxel-based architectures enable programmable deformation through controlled stiffness variation. In electronics, stretchable conductive networks maintain electrical functionality under strains exceeding several hundred per cent in hydrogel-based systems. Aerospace applications exploit multi-material architectures for lightweight structures and integrated thermal management components, where thermal conductivity enhancements and weight reductions of 10–25% have been reported relative to conventional assemblies.

These examples collectively confirm that MMAM enables spatially resolved multifunctionality unattainable through monolithic or conventionally assembled components.

5. Persistent Scientific and Industrial Challenges (Section 3.8; RQ5)

Despite substantial progress, key challenges remain. Thermal expansion mismatch, residual stress accumulation, brittle intermetallic formation, and contamination during material transitions continue to constrain reliability. Residual stresses predicted through modelling frameworks frequently approach significant fractions of material yield strength in dissimilar systems, increasing susceptibility to crack initiation.

Digital design constraints also persist. Most commercially available CAD and simulation platforms lack robust capability for modelling graded material distributions, anisotropic behaviour, and coupled thermo-mechanical interface responses. Furthermore, the absence of standardised testing protocols limits quantitative benchmarking across studies and impedes certification in safety-critical sectors.

Scalability remains an unresolved issue. While densities above 98% are routinely achievable at laboratory scale, maintaining consistent microstructural quality, dimensional stability, and interfacial integrity in high-throughput or large-format systems remains challenging.

6. Future Research Directions and Knowledge Gaps (RQ5)

Future progress in MMAM requires integrated system-level strategies combining materials science, machine engineering, computational modelling, and digital workflow development. Priority research directions include:

- Multi-physics modelling frameworks coupling phase transformation, fluid flow, and thermo-mechanical stress prediction.
- Standardised quantitative interfacial testing methodologies.
- Sustainable material handling and contamination mitigation strategies.
- Multi-material CAD and topology optimisation tools capable of predictive gradient design.
- Long-term reliability studies address fatigue, creep, thermal cycling, and environmental degradation.

Continued interdisciplinary collaboration will be essential to transition MMAM from emerging technological capability to robust industrial standard, ensuring reproducible performance, scalable production, and reliable long-term functionality.

## Figures and Tables

**Table 1:** PICOC RQ’s.

ID	Research Question
RQ1	What are the predominant interfacial, microstructural, thermal, and mechanical degradation mechanisms affecting the structural integrity and functional performance of multi-material additively manufactured components?
RQ1.1	How do these degradation mechanisms differ across polymer–polymer, metal–metal, metal–ceramic, and metal–polymer material systems fabricated using different multi-material AM processes?
RQ1.2	What material properties and microstructural features (e.g., phase distribution, porosity, grain morphology, and compositional gradients) most strongly influence interfacial strength, thermal stability, and mechanical reliability in multi-material AM parts?
RQ2	Which multi-material fabrication and interface-engineering strategies, such as functionally graded materials, in situ material mixing, hybrid deposition, and surface or interlayer modification techniques, demonstrate the greatest effectiveness in improving bonding, performance, and durability?
RQ2.1	How do different material transition architectures (e.g., discrete interfaces, graded zones, voxel-based distributions, and layered gradients) compare in terms of mechanical strength, thermal resistance, electrical/functional performance, and failure behaviour?
RQ2.2	What are the principal factors governing interface adhesion, residual stress development, defect formation, and long-term stability under cyclic thermal, mechanical, or environmental loading?
RQ3	How do process parameters, machine architectures, and hybrid manufacturing strategies influence material compatibility, interface quality, dimensional accuracy, and overall performance of multi-material AM components?
RQ3.1	Which process-specific approaches (e.g., multi-nozzle extrusion, laser power modulation, powder feeding strategies, and material switching protocols) are most effective for controlling composition, microstructure, and interfacial integrity in MMAM?
RQ3.2	How do different fabrication pathways, including standalone AM, hybrid AM–subtractive manufacturing, and functionally graded AM, affect scalability, repeatability, and post-processing requirements?
RQ4	What recent advances in design methodologies, digital workflows, and computational modelling contribute to improving material distribution control, interface prediction, and performance optimisation in multi-material AM?
RQ4.1	How do design innovations (e.g., voxel-based modelling, gradient-based material assignment, and multi-material topology optimisation) impact stress distribution, thermal behaviour, and functional integration in printed components?
RQ4.2	What limitations and practical challenges hinder the broader adoption of advanced multi-material CAD, simulation, and process-planning tools in industrial AM environments?
RQ5	What are the emerging research trends, unresolved challenges, and promising future directions for advancing the reliability, scalability, and industrial adoption of MMAM?
RQ5.1	Which novel material systems, interface architectures, and hybrid process concepts show the greatest potential for next-generation multifunctional and high-performance MMAM components?
RQ5.2	What critical knowledge gaps remain in the scientific understanding of multi-material interface behaviour, long-term performance, sustainability, and standardisation in AM?

**Table 2:** A combination of materials and processes.

	Materials	Process	Ref.
Polymers and composites	PLA	FDM	[30]
	PLA	FDM	[31]
	PLA + Carbon Fibres	FDM	[29]
	ABS + Copper	FDM	[32]
	PCL + TCP	FDM	[33]
	ABS + Al and Al_2_0_3_	FDM	[34]
	ABS + BaTi03 and PP + CaTiO_3_	FDM	[35]
	UV resin + Al_2_0_3_	SLA	[36]
	Resin-based acrylate + Microparticles of diamond	DLP	[37]
	ABS + TPU	FDM	[38]
	Nylon + Carbon Fibres	FFF	[39]
	PC/ABS + PE	FFF	[40]
	ABS + HIPS	FDM	[41]
	TPE + PA12	SLS	[42]
Metal–Metal	Stel 316L + CuSn10	SLM	[20]
	Fe + Al-12Si	SLM	[24]
	Incotel718 + Ti6Al4v	LENS™	[43]
	Ti6Al4V + CoCrMo	LENS™	[44]
	Steel 316L + Steel 430	DED	[45]
	Ti5Al2.5Sn + Ti6Al4v	LPBF	[46]
	Zinc + Copper	Fusion bonding	[47]
Metal–Ceramics	Steel 420 + TiC	SLM	[48]
	Steel 420 + TiN	SLM	[49]
	Steel 316 + WC-12%Co	LC	[50]
	AZ91D+Al+SiC	LC	[51]
	Ti6Al4V +TiC	LMD	[52]
	Steel 316 + BN	LENS™	[28]
	Steel 316 + YS-Zr	LENS™	[53]
	Titanium + zirconia	LENS™	[44]
	Al_2_O_3_ + Cu-O	LOM	[54]
Metal–Polymers	Metal, Plastic and Rubber	DIW	[55]
	Ti6Al4V + IN718	LPBF	[43]
	Maraging Steel + PLA	ME	[56]
	316L + PA11	LBF	[57]
Others	Wood + polymer	Hybrid	[58]
	B4C + SiC	DIW	[59]

**Table 3:** DED process [1,80,81,82,83,84,85,86,87,88,89].

Process	Designation	Characteristics
DLD	Direct Laser Deposition	Closed chamber with controlled atmosphere or under vacuum
DLF	Direct Light Fabrication	Inert gas
DMD	Laser-aided direct-metal/material deposition	Inert gas flow (argon) to create a protective atmosphere
LAMP	Laser-aided manufacturing process	Inert gas (argon)
Lasform	Laser forming	Argon or nitrogen gas–inert gas
LBMDMD	Laser-based multi-directional metal deposition	
LC	Laser cladding	Shielding Gas (Ar, He)
LDT	Laser deposition technology	Controlled atmosphere
LENS™	Laser-engineered net shaping	Controlled atmosphere
LMD	Laser metal deposition	protective atmosphere
LPF	Laser powder fusion	
SDM	Shape deposition manufacturing	Inert gas

**Table 4:** Author-synthesised comparison of major processing routes for FGMs, organised by characteristics.

Process Family	Processing Route	Gradient Control Mode	Characteristic Build Scale *	Compositional Latitude	Typical Product Form	Geometric Suitability
Powder/layer assembly	Powder stacking	Layerwise, stepwise	0.1 mm to >1 mm	Broad	Bulk components	Moderate
Powder/layer assembly	Sheet lamination	Layerwise, stepwise	10–1000 μm	Broad	Bulk components	Moderate
Suspension/coating routes	Wet powder spraying	Near-continuous to stepwise	<10–100 μm	Broad	Bulk preforms	Moderate
Suspension/coating routes	Slurry dipping	Continuous through repeated immersion	<10–100 μm	Broad	Surface layers/coatings	High
Melt/deposition routes	Jet solidification	Dynamically controlled during deposition	0.1 mm to >1 mm	Broad	Bulk components	Very high
Particle rearrangement routes	Sedimentation/centrifugation	Continuous through particle redistribution	Continuous	Broad	Bulk components	Low
Particle rearrangement routes	Filtration/slip casting	Continuous through filtration gradient	Continuous	Broad	Bulk components	High
Melt/deposition routes	Laser cladding	Dynamically controlled during deposition	~0.1–1 mm	Broad	Bulk build-up/coatings	Very high
Melt/deposition routes	Thermal spraying	Layerwise or quasi-continuous	10–100 μm	Broad	Coatings/thick surface layers	High
Diffusion-driven routes	Diffusion bonding/grading	Continuous through interdiffusion	Continuous	Broad	Joints/coatings	High
Solidification-controlled routes	Directional solidification	Continuous through thermal field control	Continuous	Moderate	Bulk components	Low
Electrochemical routes	Electrochemical gradation	Continuous through electrochemical control	Continuous	Moderate to broad	Bulk components	High
Polymer processing routes	Polymer foaming	Continuous through density/composition variation	Continuous	Moderate to broad	Bulk components	High
Vapour-phase routes	PVD/CVD	Continuous at surface scale	Continuous, thin-film regime	Broad	Coatings	Moderate
Specialised composite routes	GMFC-based processing	Stepwise or semi-continuous	0.1 mm to >1 mm, or continuous depending on route	Moderate	Bulk components	High

* Build scale categories are expressed here as practical ranges rather than ranking terms in order to provide a process-oriented comparison. The table is presented as an original synthesis for comparative review purposes, rather than as a reproduced classification scheme.

**Table 5:** Different parameters of AM [1,117].

AM-Related Powdered Milk-Based Processes	Fused Deposition Modelling
Laser power Scan spacing Scanning speed Deposition strategy Layer thickness Powder size Spot diameter	Build orientation Layer thickness Scan angle/orientation Extrusion/deposition nozzle diameter Printing speed Extrusion temperature Platform temperature Infill density and pattern/style Scanning width Gap/empty space Scanning orientation Contour thickness

**Table 6:** Comparative Performance of MMAM Material Systems and Processes [18,122,158,159,160].

Material System	AM Process	Typical Interfacial Strength	Thermal Conductivity Effect	Residual Stress Level	Key Advantage	Technology Maturity
Polymer–Polymer	FDM/DIW	10–35 MPa	Low–Moderate	Low	Good compatibility	High
Polymer–Composite	FDM/FFF	25–70 MPa	Improved stiffness	Low–Moderate	Lightweight structures	High
Metal–Metal	SLM/LPBF	150–400 MPa	High	High	Structural performance	Medium–High
Metal–Ceramic	LENS/DED	80–250 MPa	Very high	Very High	Wear & thermal resistance	Medium
Metal–Polymer	Hybrid AM	20–120 MPa	Moderate	Interface sensitive	Functional integration	Emerging
Functionally Graded Materials	LPBF/DED	Reduced interface failure	Optimised heat transfer	Reduced vs discrete	Stress mitigation	Emerging–Advanced

**Figure A1:**
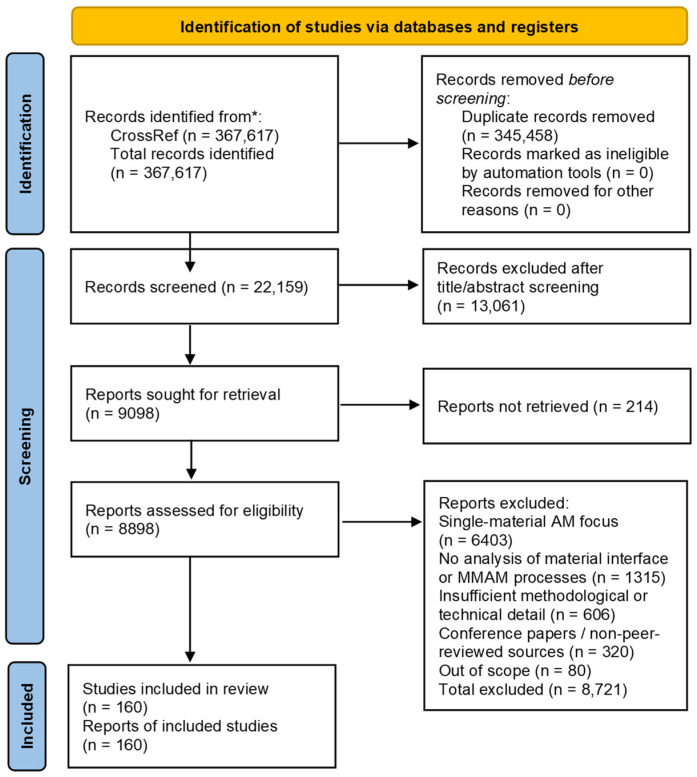
PRISMA 2020 flowchart for the MMAM study.

## Data Availability

No new data were created or analyzed in this study.

## Appendix A

### PRISMA 2020 Flow Diagram for New Systematic Reviews, Which Included Searches of DataBases and Registers

This appendix provides the detailed PRISMA 2020 flow diagram describing the study identification, screening, eligibility assessment, and inclusion process undertaken in this systematic review. The diagram ensures transparency and reproducibility of the literature selection methodology in accordance with the PRISMA 2020 statement.

The systematic search strategy was conducted across multiple scientific databases and relevant registers to identify peer-reviewed literature related to MMAM. The search combined predefined keywords associated with multi-material processing, interface behaviour, graded materials, and additive manufacturing technologies. Boolean operators and controlled vocabulary terms were applied where appropriate to maximise sensitivity and specificity. The selection process is depicted in the flowchart of Figure 10.
